# In-Person and Telehealth Ambulatory Contacts and Costs in a Large US Insured Cohort Before and During the COVID-19 Pandemic

**DOI:** 10.1001/jamanetworkopen.2021.2618

**Published:** 2021-03-23

**Authors:** Jonathan P. Weiner, Stephen Bandeian, Elham Hatef, Daniel Lans, Angela Liu, Klaus W. Lemke

**Affiliations:** 1 Center for Population Health Information Technology, Department of Health Policy and Management, Johns Hopkins Bloomberg School of Public Health, Baltimore, Maryland; 2 Blue Health Intelligence, LLC, an independent licensee of the Blue Cross and Blue Shield Association, Chicago, Illinois

## Abstract

**Importance:**

This study assesses the role of telehealth in the delivery of care at the start of the COVID-19 pandemic.

**Objectives:**

To document patterns and costs of ambulatory care in the US before and during the initial stage of the pandemic and to assess how patient, practitioner, community, and COVID-19–related factors are associated with telehealth adoption.

**Design, Setting, and Participants:**

This is a cohort study of working-age persons continuously enrolled in private health plans from March 2019 through June 2020. The comparison periods were March to June in 2019 and 2020. Claims data files were provided by Blue Health Intelligence, an independent licensee of the Blue Cross and Blue Shield Association. Data analysis was performed from June to October 2020.

**Main Outcomes and Measures:**

Ambulatory encounters (in-person and telehealth) and allowed charges, stratified by characteristics derived from enrollment files, practitioner claims, and community characteristics linked to the enrollee’s zip code.

**Results:**

A total of 36 568 010 individuals (mean [SD] age, 35.71 [18.77] years; 18 466 557 female individuals [50.5%]) were included in the analysis. In-person contacts decreased by 37% (from 1.63 to 1.02 contacts per enrollee) from 2019 to 2020. During 2020, telehealth visits (0.32 visit per person) accounted for 23.6% of all interactions compared with 0.3% of contacts in 2019. When these virtual contacts were added, the overall COVID-19 era patient and practitioner visit rate was 18% lower than that in 2019 (1.34 vs 1.64 visits per person). Behavioral health encounters were far more likely than medical contacts to take place virtually (46.1% vs 22.1%). COVID-19 prevalence in an area was associated with higher use of telehealth; patients from areas within the top quintile of COVID-19 prevalence during the week of their encounter were 1.34 times more likely to have a telehealth visit compared with those in the lowest quintile (the reference category). Persons living in areas with limited social resources were less likely to use telehealth (most vs least socially advantaged neighborhoods, 27.4% vs 19.9% usage rates). Per enrollee medical care costs decreased by 15% between 2019 and 2020 (from $358.32 to $306.04 per person per month). During 2020, those with 1 or more COVID-19–related service (1 470 721 members) had more than 3 times the medical costs ($1701 vs $544 per member per month) than those without COVID-19–related services. Persons with 1 or more telehealth visits in 2020 had considerably higher costs than persons having only in-person ambulatory contacts ($2214.10 vs $1337.78 for the COVID-19–related subgroup and $735.87 vs $456.41 for the non–COVID-19 subgroup).

**Conclusions and Relevance:**

This study of a large cohort of patients enrolled in US health plans documented patterns of care at the onset of COVID-19. The findings are relevant to policy makers, payers, and practitioners as they manage the use of telehealth during the pandemic and afterward.

## Key Points

**Question:**

How did ambulatory care patterns change after the initial COVID-19 surge in the US and what role did telehealth play?

**Findings:**

In this cohort study of 36 568 010 US individuals, ambulatory contacts decreased by 18% between the 2019 and 2020 March to June periods, and telehealth use increased from 0.3% of contacts in 2019 to 23.6% of all contacts in 2020. Increased disease burden, COVID-19 prevalence, and greater social resources were associated with higher telehealth use.

**Meaning:**

These findings suggest that the dramatic shift in the adoption of virtual care has many implications for health services provision both during and after the pandemic.

## Teaser

This cohort study examines the patterns and costs of ambulatory care in the US before and during the initial stage of the COVID-19 pandemic and assesses how patient, practitioner, community, and COVID-19–related factors are associated with telehealth adoption.

## Introduction

The use of telehealth services during the COVID-19 pandemic has been unprecedented. During the initial March to April 2020 peak, case reports suggested that telehealth usage went from less than 1% of visits^1^ to as much as 80% where COVID-19 prevalence was high.^2^

Although telehealth services have been available in the US for decades, its adoption was still uncommon before the COVID-19 pandemic.^3,4^ Barriers to use included limited insurance coverage and regulations regarding jurisdiction of licensure. Also, many practitioners and patients were not technically equipped to offer and use these digitally mediated services.^5^

The COVID-19 pandemic has also placed unprecedented challenges on government, practitioners, and patients to mitigate the spread of this disease. Therefore, telehealth emerged as a way to deliver care at a social distance. In response, policy makers, payers, and practitioners eliminated almost all financial, regulatory, and technical barriers that hampered past telehealth expansion.^6,7,8^

The shift to telehealth has impacted not only the more than 7.5% of Americans with confirmed COVID-19,^9^ but also every person coming in contact with the health care system. Most policy, clinical, and electronic health experts believe that although coverage policies and practitioner and consumer telehealth adoption levels may change once the pandemic subsides, the adoption trajectory of these technologies has been forever changed.^10,11,12^

This study undertakes an assessment of the shifts in telehealth and ambulatory visit use during the first 4 months of the COVID-19 pandemic (March to June 2020). We document overall ambulatory contacts and costs both before (2019) and after (2020) the pandemic’s start within a very large cohort of more than 36 million working-age, insured US individuals. We assess the associations of key patient, community, and health system characteristics with service use. We also assess the association of telehealth use with a patient having a COVID-19–related diagnosis and/or residing in a high COVID-19 prevalence area.

## Methods

### Data Sources and Cohort Selection

The institutional review board of the Johns Hopkins Bloomberg School of Public Health reviewed and approved this cohort study as being exempt; thus, informed consent was not sought. This study conforms to the Strengthening the Reporting of Observational Studies in Epidemiology (STROBE) reporting guideline.^13^

Data for this study came from the Blue Health Intelligence data repository. Blue Health Intelligence is an independent data and analytics company that is a licensee of the Blue Cross and Blue Shield Association. This database is sourced from a large number of health insurance plans. The study data set included complete claims files for commercial health plan members continuously enrolled from March 1, 2019, through June 30, 2020. Claims submitted through July 31, 2020, were included. eFigure 1 in the Supplement provides information on sample selection and subgroup identification. Distribution of the study population by US Census region is presented in eTable 1 in the Supplement.

Our analysis compared the 4-month period of March through June in 2019 and 2020 to account for seasonality. The study population was limited to persons covered through employer-based, Affordable Care Act, and other private health insurance plans, but not Medicare or Medicaid.

We developed an approach to identify persons treated for actual or suspected COVID-19. We designated a person to this COVID-19–related subgroup if they had any diagnosis code associated with COVID-19 disease, exposure, screening, or testing during the 2020 study period. eTable 2 in the Supplement presents identification criteria and study member counts for the COVID-19–related subgroup.

### Definition of Ambulatory and Telehealth Encounters

Our unit of analysis was an ambulatory encounter with a telehealth-eligible service. Inpatient and emergency department services were excluded. An encounter was defined as a patient seeing a specific practitioner on a specific date. Telehealth-eligible services were identified using *Current Procedural Terminology* or *Healthcare Common Procedure Codes* that, on the basis of payer policy, were eligible for telehealth coverage (see eTable 3 and eTable 4 in the Supplement).^14^ Telehealth-eligible services were subdivided between those provided in person or via telehealth. Some telehealth-eligible codes can only be used for telehealth, whereas others may be provided either way. Thus, we classified them as telehealth only when an appropriate modifier was present (see eTable 5 in the Supplement). In accordance with payment policy, we considered telehealth to include any service provided on a remote basis, whether via video, telephone, asynchronous or synchronous text or email transmission, or other similar technology. Details on designation of telehealth modality are provided in eTable 5 in the Supplement.

Some encounters include multiple claim lines (mean [SD], 1.13 [0.58] claim lines). To assign encounter-level attributes (eg, diagnosis, specialty), we selected a principal service for each unique encounter based on the claim line with the highest allowed charge. The categorization we used to assign a specialty to each practitioner is described in eTable 6 in the Supplement.

### Study Member Characteristics and Definition of Measures

We captured information on the members’ demographic characteristics, including age, sex, and zip code of residence from enrollment files. Using zip code, we mapped each study member to a US Census region, state, and county and to urban or rural categorizations.^15^ We assigned a member’s residence to 1 of 4 levels of social deprivation based on a national ranking of the Area Deprivation Index.^16^ For person-oriented analyses, we classified a residence as a COVID-19 hot spot if their state had a prevalence greater than or equal to 1.5 times the national mean on May 1, 2020.^17^ For encounter-level analyses, we assigned the national decile rank of prevalence for the patient’s zip code during the week of service.^18^

We documented each member’s chronic condition count (0, 1, 2, or ≥3 conditions) according to all ambulatory care primary diagnoses noted during the 12 months of 2019.^19^ We also categorized the study member’s type of insurance as standard preferred provider organization, high-deductible plan (ie, a deductible >$1000), or health maintenance organization.

Finally, we classified each encounter as involving a new patient, if the enrollee had not visited the billing practitioner organization within the past 3 years and as a new condition if there had not been a visit for that primary diagnosis within the past 3 years.^20^ All members of the cohort were continuously enrolled at least from March 2019, but for this continuity of care assessment, we reviewed data from 2016 forward for those members who were enrolled longer.

### Service Costs

To assess health care costs, we analyzed allowed charges. This corresponded to the dollar amount allowed by plan fee schedules before any enrollee cost sharing. For comparability, 2019 charges were adjusted upward to reflect 2020 pricing using relevant components of the Consumer Price Index (1.4% to 3.7% depending on service type).^21^

To account for services provided before the end of June 2020, but where claims were not submitted or closed as of July 31, 2020 (our data set creation date), completion factors were applied on the basis of 2018 patterns. These lag adjustments for 2020 equaled an upward adjustment of 12.8% for allowed charges and 5.1% for encounters.

### Statistical Analysis

Log-binomial multivariable logistic regression was used to calculate relative risk ratios (RRRs) for member-specific and encounter-specific factors to assess their independent associations with the likelihood of telehealth vs in-person contacts in 2020. Significance was set at 2-sided *P* < .05. Only persons with 1 or more telehealth-eligible service were included in the analysis (15 437 217 individuals). These individuals had 46 453 007 in-scope encounters. For computational efficiency, in lieu of specifying a random effect structure, robust sandwich variance estimators were used to improve estimates for standard errors, to account for potential multiple encounters.

We measured the associations of member-level characteristics on whether the member experienced 1 or more telehealth service during the study period. For encounter-level analyses, the outcome was defined as a binary (yes or no) telehealth indicator for that encounter. For both types of analyses, we used categorical variables derived from the enrollment file and factors linked to a member’s home zip code.

We used SAS statistical software version 9.4 (SAS Institute) and R statistical software version 4.0 (R Project for Statistical Computing) to conduct the analyses.^22^ Data analysis was performed from June to October 2020.

## Results

In this cohort study of 36 568 010 individuals (mean [SD] age, 35.71 [18.77] years; 18 466 557 female individuals [50.5%]), ambulatory contacts decreased by 18% (from 1.64 to 1.34 visits per person) between 2019 and 2020 (Table 1). When only in-person visits were considered, the decrease equaled 37% (from 1.63 to 1.02 visits per person). The proportion of persons with at least 1 contact of any kind during each of the 4-month periods decreased by approximately 19%, from 51.8% (18 942 276 persons) in 2019 to 42.2% (15 437 217 persons) in 2020.

**Table 1.** In-Person and Telehealth Ambulatory Contacts for Continuously Enrolled Insured Study Cohort for March to June 2019 (Before the COVID-19 Pandemic) and March to June 2020 (During the COVID-19 Pandemic) Study Periods^a^

Variable	Participants, No. (%) (N = 36 568 010)	
	March-June 2019	March-June 2020
Enrollees with ≥1 ambulatory contact	18 942 276 (51.8)	15 437 217 (42.2)
Enrollees with ≥1 telehealth visit	109 704 (0.3)	4 977 415 (13.6)
Ambulatory contacts per enrollee, No.		
Total ^b^	1.64	1.34
In-person^b^	1.63	1.02
Telehealth	.01	.32
Telehealth claims submitted in 2020 by modality		
Video supported	NA	11 296 298 (74.4)
Telephonic	NA	1 391 618 (9.2)
Email, chat, or other	NA	502 837 (3.3)
Unspecified	NA	1 988 277 (13.1)
Total	NA	15 179 030 (100)

Abbreviation: NA, not applicable.

^a^ Sample included persons continuously enrolled from March 1, 2019, through June 30, 2020, within private health insurance plans in the data files of Blue Health Intelligence, an affiliate of the Blue Cross and Blue Shield Association. See further details regarding sample selection in the text and eFigure 1 and eTable 1 in the Supplement.

^b^ All results consider only ambulatory encounters that are telehealth eligible. Total contacts include both in-person and telehealth visits. Contacts per enrollee include both users and nonusers. See text and eTable 3, eTable 4, and eTable 5 in the Supplement for further details.

In 2019, 179 805 ambulatory visits (0.3%) were billed using telehealth designations. During 2020, the percentage was 23.6% (0.32 visit per person). In 2020, 13.6% of all persons (4 977 415 individuals) experienced 1 or more telehealth interactions. The trends for both total ambulatory contacts and the percentage delivered via telehealth are presented graphically week by week during 2020 in eFigure 2 and eFigure 3 in the Supplement.

On the basis of billing designations, video-supported telehealth visits represented 74.4% of the virtual visits (11 296 298 claims) (Table 1). Given that 13.1% of telehealth claims (1 988 277 claims) did not specify a modality, it is possible that the majority of this missing data group also had video visits.

Table 2 presents the unadjusted member-level rates of total and telehealth ambulatory contacts during the 2019 and 2020 periods according to member characteristics, including those linked to their zip code. Notable 2020 findings are that age and disease burden appear to be associated with telehealth uptake, with those aged 18 to 49 years and with 2 or more chronic conditions using more telehealth. Virtual care use rates were higher in states that represented COVID-19 hot spots during the study period (36.0% [1.28 contacts per person] vs 21.6% [1.35 contact per person]), urban vs rural locales (24.2% [1.35 contacts per person] vs 14.2% [1.15 contacts per person]), and in the most vs least socially advantaged neighborhoods (27.4% [1.42 contacts per person] vs 19.9% [1.24 contacts per person]). Persons in health maintenance organizations were more likely to use telehealth (35.7% [1.48 contacts per person]) compared with those in standard preferred provider organizations (24.1% [1.41 contacts per person]) or high-deductible plans (19.1% [1.14 contacts per person]). Members in the COVID-19–related subgroup had ambulatory visit rates that were approximately 30% higher (3.99 vs 3.08 contacts per person) than those without COVID-19–related diagnoses, and their use of telehealth was slightly higher (25.0% vs 23.5% of visits).

**Table 2.** Ambulatory Encounters and Percentage Telehealth for March to June 2019 (Before the COVID-19 Pandemic) and March to June 2020 (During the COVID-19 Pandemic) By Presence of COVID-19–Related Diagnoses and Characteristics of Patients and Their Residence

Patient level characteristics	Patients, No. (%) (N = 36 568 010)^a^	Contacts per person, No. (% that were telehealth)			
		March-June 2019, all persons^a^	March-June 2020		
			All persons^a^	Persons with ≥1 contact, not in COVID-19–related subgroup (n = 13 966 497)	Persons with ≥1 contact, in COVID-19–related subgroup (n = 1 470 721)^b^
Column mean		1.64 (0.3)	1.34 (23.6)	3.08 (23.5)	3.99 (25.0)
Chronic conditions treated in 2019					
0	20 420 796 (55.8)	0.58 (0.4)	0.58 (17.4)	2.16 (16.9)	2.58 (20.8)
1	8 804 381 (24.1)	1.91 (0.3)	1.52 (24.2)	2.83 (24.1)	3.60 (24.9)
2	4 132 359 (11.3)	3.21 (0.3)	2.47 (26.2)	3.53 (26.2)	4.63 (26.6)
≥3	3 210 474 (8.8)	5.58 (0.3)	4.18 (26.7)	4.87 (26.5)	6.82 (27.4)
Age in 2019, y					
0-5	2 451 286 (6.7)	1.74 (0.1)	0.84 (16.4)	2.31 (16.2)	3.18 (19.4)
6-17	5 722 334 (15.6)	1.41 (0.2)	0.98 (25.7)	3.03 (25.8)	3.14 (25.3)
18-34	9 139 263 (25.0)	1.28 (0.5)	1.15 (28.1)	3.09 (28.3)	3.43 (27.4)
35-49	8 911 461 (24.4)	1.62 (0.4)	1.37 (25.4)	3.00 (25.2)	3.88 (26.7)
50-64	9 594 054 (26.2)	2.04 (0.2)	1.73 (20.2)	3.17 (19.8)	4.60 (22.8)
≥65	749 612 (2.0)	2.50 (0.1)	2.46 (19.5)	4.43 (19.1)	6.85 (22.2)
Residence in COVID-19 hot spot^c^					
No	31 146 733 (85.2)	1.60 (0.3)	1.35 (21.6)	3.04 (21.5)	3.95 (22.7)
Yes	5 421 277 (14.8)	1.86 (0.2)	1.28 (36.0)	3.30 (36.1)	4.22 (35.4)
US Census region of residence					
Northeast	6 940 071 (19.0)	1.79 (0.2)	1.19 (35.5)	3.11 (35.2)	4.13 (37.6)
Midwest	9 309 546 (25.4)	1.62 (0.3)	1.35 (21.6)	3.23 (21.5)	4.31 (22.7)
South	15 490 398 (42.4)	1.59 (0.4)	1.39 (20.1)	2.92 (20.0)	3.74 (21.0)
West	4 827 995 (13.2)	1.61 (0.4)	1.35 (24.1)	3.32 (24.0)	4.21 (24.9)
Urban vs rural residence					
Urban	34 229 907 (93.6)	1.66 (0.3)	1.35 (24.2)	3.11 (24.0)	4.01 (25.6)
Rural	2 338 103 (6.4)	1.36 (0.5)	1.15 (14.2)	2.63 (14.2)	3.68 (14.6)
Area Deprivation Index of residence by quartile^d^					
1 (Low)	14 818 612 (40.5)	1.83 (0.3)	1.42 (27.4)	3.35 (27.4)	4.22 (28.1)
2	8 368 751 (22.9)	1.57 (0.3)	1.29 (21.9)	2.98 (21.7)	3.95 (23.5)
3	7 498 625 (20.5)	1.51 (0.3)	1.27 (20.0)	2.86 (19.8)	3.83 (21.7)
4 (High)	5 547 985 (15.2)	1.41 (0.3)	1.24 (19.9)	2.79 (19.4)	3.69 (22.8)
Type of insurance plan in 2019					
Standard	23 962 063 (65.5)	1.73 (0.3)	1.41 (24.1)	3.19 (23.9)	4.13 (25.5)
Health maintenance organization	2 116 563 (5.8)	1.74 (0.2)	1.48 (35.7)	3.20 (35.3)	4.16 (38.1)
High deductible	10 489 384 (28.7)	1.42 (0.3)	1.14 (19.1)	2.77 (19.1)	3.59 (19.7)

^a^ The entire sample (of users and nonusers) included 36 568 010 persons continuously enrolled in commercial insurance plans from March 1, 2019, through June 30, 2020. These unadjusted rates are reported as number of telehealth eligible ambulatory contacts per enrollee and percentage of these contacts that took place via telehealth during each 4-month study period (March-June). All rows are calculated at the person level. See text for further details.

^b^ Refers to 1 or more COVID-19–related diagnosis codes reported for each person. Refer to eTable 2 in the Supplement for more information on the methods used to identify persons with potential or actual COVID-19 diagnosis during 2020 study period. This includes people tested and/or treated and/or diagnosed with COVID-19.

^c^ A COVID-19 hot spot is defined as states that on May 1, 2020, had a COVID-19 case count per 100 000 residents greater than or equal to 1.5 times the national mean (see text for additional details).

^d^ Area Deprivation Index is a social deprivation measure based on a national population-weighted ranking. The higher the number, the more deprived the community (see text for additional details).^15^ Note that for less than 1% of the sample it was not possible to assign an Area Deprivation Index Score. Weighted rankings are shown.

Table 3 presents unadjusted 2019 and 2020 encounter-level analyses stratified by visit characteristics. Note that unlike overall encounters, which decreased, the per member rate for behavioral visits stayed constant. Mental health visits were far more likely than medical office visits to be delivered via telehealth (46.1% [0.23 visit per person] vs 22.1% [0.86 visit per person]). There was variability in telehealth use by clinical specialty and practitioner type; surgical and rehabilitation services (including physical therapy) were far less likely than other types of services to be delivered virtually (9.2% [0.10 visit per person] and 4.6% [0.15 visit per person], respectively). It appears that the total encounter rate (in-person and telehealth combined) addressing diabetes, hypertension, cancer, and well-child care stayed almost the same from 2019 to 2020. The greatest per member decrease, from 0.89 to 0.63 visit per person, occurred for visits with acute primary diagnoses. Telehealth usage for acute conditions (14.1% [0.63 visit per person]) was far lower than that for chronic condition visits (21.5% [0.24 visit per person]). The use of telehealth for new patients and for new problems in 2020 was lower than overall rates.

**Table 3.** Ambulatory Encounters and Percentage Telehealth Contacts for March to June 2019 (Before the COVID-19 Pandemic) and March to June 2020 (During the COVID-19 Pandemic) by Presence of COVID-19–Related Diagnoses and Characteristics of the Visit or Encounter

Encounter level characteristics	Contacts per person, No. (% that were telehealth)			
	March-June 2019, all persons (N = 36 568 010)^a^	March-June 2020		
		All persons^a^	Persons with ≥1 contact, not in COVID-19–related subgroup (n = 13 966 497)	Persons with ≥1 contact, in COVID-19–related subgroup (n = 1 470 721)^b^
Column mean for entire subgroup	1.64 (0.3)	1.34 (23.6)	3.08 (23.5)	3.99 (25.0)
Type of ambulatory contact				
Evaluation and management office visit	1.06 (0.3)	0.86 (22.1)	1.93 (21.7)	2.99 (24.4)
Behavioral health	0.23 (0.4)	0.23 (46.1)	0.56 (45.8)	0.47 (49.1)
Rehabilitation	0.25 (0.0)	0.17 (2.4)	0.41 (2.4)	0.40 (1.7)
Other	0.09 (0.8)	0.07 (20.5)	0.17 (20.2)	0.13 (25.3)
Practitioner specialty				
Primary care MD or DO	0.47 (0.3)	0.37 (24.0)	0.84 (23.1)	1.28 (29.6)
Medical specialist MD or DO	0.14 (0.1)	0.12 (24.9)	0.29 (24.5)	0.36 (27.9)
Surgical specialist MD or DO	0.14 (0.0)	0.10 (9.2)	0.23 (8.9)	0.30 (11.8)
Behavioral health MD, DO, or non-MD	0.12 (0.4)	0.12 (44.9)	0.28 (44.6)	0.25 (48.0)
Rehabilitation MD, DO, or non-MD	0.22 (0.0)	0.15 (4.6)	0.36 (4.7)	0.32 (4.3)
Other physician	0.33 (0.7)	0.30 (30.6)	0.70 (31.1)	0.87 (26.6)
Physician assistant or nurse practitioner	0.13 (0.3)	0.11 (19.4)	0.24 (19.2)	0.38 (20.5)
Other nonphysician	0.09 (0.4)	0.07 (22.0)	0.15 (22.5)	0.24 (19.2)
Primary diagnosis				
Diabetes	0.04 (0.2)	0.03 (24.7)	0.08 (24.5)	0.07 (27.5)
Hypertension	0.05 (0.1)	0.04 (26.4)	0.10 (26.2)	0.09 (28.5)
Behavioral health	0.33 (0.5)	0.33 (44.7)	0.79 (44.4)	0.64 (48.1)
Cancer	0.03 (0.1)	0.03 (13.7)	0.06 (13.2)	0.12 (15.9)
Well child-care	0.01 (0.0)	0.01 (1.1)	0.02 (1.1)	0.01 (2.1)
COVID-19 diagnosis	0.00 (0.0)	0.03 (22.1)	0.00 (0.0)	0.62 (22.1)
Other chronic diagnosis	0.30 (0.1)	0.24 (21.5)	0.56 (21.2)	0.64 (23.7)
Other acute diagnosis	0.89 (0.3)	0.63 (14.1)	1.46 (13.5)	1.80 (18.7)
Continuity of patient or problem				
New patient encounters	0.36 (0.5)	0.22 (15.7)	0.47 (15.6)	0.93 (15.8)
New problem encounters	0.56 (0.4)	0.38 (18.9)	0.82 (18.2)	1.55 (22.0)

^a^ The full study sample included persons continuously enrolled from March 1, 2019, through June 30, 2020. These unadjusted rates are reported as number of telehealth eligible ambulatory contacts per enrollee that took place during each 4-month (March-June) study period. Percentages represent proportion of contacts that took place via telehealth. Note that all rows were calculated at encounter level and that the “all persons” columns include all enrollees whether or not they used any services. See text for further details.

^b^ Refers to 1 or more COVID-19–related diagnosis codes reported for each person. Refer to eTable 2 in the Supplement for more information on the methods used to identify persons with potential or actual COVID-19 diagnosis during 2020 study period. This includes people tested and/or treated and/or diagnosed with COVID-19.

Table 4 presents mean per member per month (PMPM) allowed charges. This is a standard measure of a group’s resource consumption used by the insurance industry. When assessing the entire population of both users and nonusers, total medical costs decreased by approximately 15% between 2019 and 2020 (from $358.32 to $306.04 PMPM). Even as costs for the total study cohort decreased, medical expenditures for those with 1 or more ambulatory contact increased modestly from $632.48 to $653.78 PMPM.

**Table 4.** Cost of Care (per Member per Month) During March to June 2019 (Before the COVID-19 Pandemic) and 2020 (During the COVID-19 Pandemic) by Ambulatory or Telehealth Use Categories and Presence or Absence of COVID-19–Related Diagnosis

Cost category	March-June 2019		March-June 2020					
	Persons with all diagnoses		Persons with all diagnoses		Not in COVID-19–related subgroup^a^		COVID-19–related subgroup^a^	
	All persons	Persons with ≥1 contact	All persons	Persons with ≥1 contact	Persons with ≥1 in-person contact (no telehealth)	Persons with ≥1 telehealth contact	Persons with ≥1 in-person contact (no telehealth)	Persons with ≥1 telehealth contact
Enrollees, No. (% of study cohort)^b^	36 568 010 (100)	18 942 276 (51.8)	36 568 010 (100)	15 437 217 (42.2)	9 591 554 (26.2)	4 374 943 (11.9)	868 249 (2.4)	602 472 (1.7)
Medical costs per member per month in 2020 $US^c^								
Inpatient^d^	77.51	133.39	76.90	158.23	92.91	165.24	410.36	783.86
Total outpatient^e^	280.82	499.09	229.15	495.55	363.50	570.63	927.43	1430.24
Emergency department	13.29	21.25	9.36	17.85	13.50	16.04	46.10	59.47
Ambulatory visits	49.18	94.79	39.81	94.26	70.64	136.92	87.09	170.99
In-person	49.09	94.62	30.85	73.03	70.61	71.58	87.05	101.80
Via telehealth	0.09	0.18	8.96	21.24	0.02	65.34	0.04	69.20
All other outpatient^f^	218.35	383.05	179.98	383.44	279.36	417.67	794.24	1199.78
Total medical costs	358.32	632.48	306.04	653.78	456.41	735.87	1337.78	2214.10
Pharmacy costs per member per month in 2020 $US^g^	126.32	210.12	139.36	260.30	199.46	384.62	195.09	424.32

^a^ Refers to 1 or more COVID-19–related diagnosis codes reported for each person. Refer to eTable 2 in the Supplement for more information on the methods used to identify persons with potential or actual COVID-19 diagnosis during the 2020 study period. This includes people tested and/or treated and/or diagnosed with COVID-19. Also see eTable 2 in the Supplement for breakdown of hospitalization status in COVID-19–related subgroup.

^b^ The full study sample consists of continuously enrolled health plan members from March 1, 2019, through June 30, 2020. Note the numbers and percentages in the first row are based on the full study cohort for which medical services were analyzed. As noted, the prescription costs in the bottom row were calculated for a subset of this cohort.

^c^ Cost figures reflect per enrollee or member per month allowed charges calculated over the four-month (March-June) study periods in each year; 2019 dollars were inflated to 2020 dollars.

^d^ Inpatient included all facility and professional services included in inpatient stays.

^e^ Outpatient included all other facility, professional, and supplier services with a breakdown by subcategory. Emergency department included both facility and professional services.

^f^ Includes ambulatory surgery, imaging, laboratory, and chemotherapy.

^g^ The pharmacy row shows data for the subset of 20 131 369 persons with full prescription coverage during the entire study period.

Overall, 1 470 721 members in the COVID-19–related subgroup (with or without telehealth) had more than 3 times higher medical costs than the non–COVID-19 user subgroup ($1701 vs $544 PMPM). The COVID-19–related subgroup was somewhat heterogeneous, including both persons being screened and treated for coronavirus infection. eTable 2 in the Supplement provides further details on their distribution of COVID-19–related diagnoses and hospitalizations.

As described in Table 4, for both the subgroups with and without COVID-19–related diagnoses, persons with 1 or more telehealth contacts had considerably higher medical costs than those without telehealth visits ($2214.10 vs $1337.78 for the COVID-19–related subgroup and $735.87 vs $456.41 for the non–COVID-19 subgroup). eTable 7 in the Supplement compares the breakdown of age and chronic disease among the 2020 subgroups and indicates that persons with 1 or more telehealth visit were older and had greater preexisting disease burden than those with in-person visits only.

For the entire (user and nonuser) study population, pharmacy costs increased slightly across the periods (from $126.32 in 2019 to $139.36 PMPM in 2020). Unlike medical costs, pharmacy costs were not appreciably higher among the COVID-19–related subgroup.

The Figure presents RRRs at either the encounter or member level as appropriate. These reflect the adjusted probability that in 2020 a user will be seen by telehealth vs in-person, given 4 key factors: COVID-19 prevalence in their community during the week of the encounter, patient’s preexisting chronic disease burden, primary diagnosis assigned to the encounter, and urban designation of the patient’s residence. Each plot shows whether the variable of interest was associated with higher (>1.0), lower (<1.0), or equal (1.0) likelihood of a telehealth visit compared with the telehealth use for patients within the benchmark reference category, holding constant the other variables noted. For example, encounters for patients from areas within the top quintile of COVID-19 prevalence during the week of their encounter were 1.34 times more likely to have a telehealth visit compared with those in the lowest quintile (the reference category), all else being equal.

**Figure.**
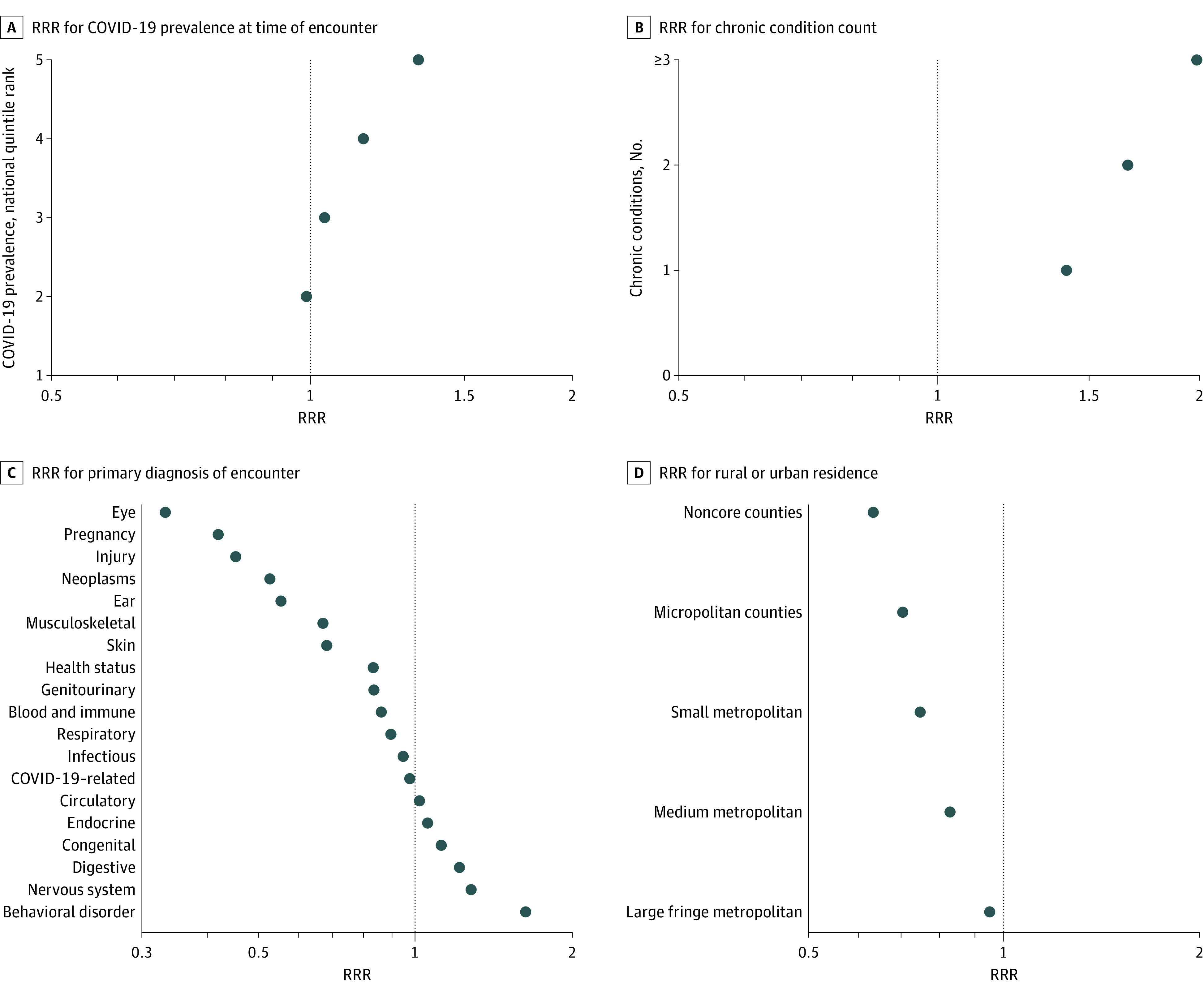
Adjusted Relative Risk Ratios (RRRs) Associated With Telehealth vs In-Person Ambulatory Visits During 2020 COVID-19 Study Period The RRRs reflect the odds of a telehealth contact vs in-person visit. The sample included 15 427 217 persons in the continuously insured cohort with 1 or more telehealth eligible ambulatory visit during the 4-month March to June 2020 study period. These individuals had 46 453 007 in-scope encounters. All RRRs are adjusted for age, sex, plan type, chronic condition count, morbidity categories, region, urban vs rural residence, Area Deprivation Index, COVID-19 prevalence during week (for encounter) or hot spot (for person level), and type of service. For panel A, the reference is the first (lowest) quintile. For panel B, the reference is 0 chronic conditions. For panel C, the reference is *International Statistical Classification of Diseases and Related Health Problems, Tenth Revision *diagnosis chapter for signs and symptoms. For panel D, the reference is large central metropolitan area. The encounter-level analyses (panels A and B) are also adjusted for practitioner specialty and new patient and new condition combinations for that contact. RRR values are plotted on log scales. Every result shown here is statistically significant (*P* < .05) and does not overlap with an RRR of 1.0 because of the large sample size. See eFigure 4 in the Supplement for the numeric value of each RRR and 95% CIs, along with additional adjusted RRR analysis results for other key variables.

In addition to the apparent influence of COVID-19 prevalence, these multivariable analyses showed a distinct pattern of RRRs across diagnoses, with eye, pregnancy, injury, and cancer encounters most likely to be in person. Encounters with behavioral diagnoses were far more likely to be virtual. Additionally, persons with greater chronic disease burden (in 2019) were considerably more likely to get care via telehealth during 2020. The Figure indicates that all else equal, the more rural the patient’s home jurisdiction, the less likely they were to have at least 1 telehealth contact.

eFigure 4 in the Supplement presents 95% CIs and numeric results for each RRR analysis presented in the Figure. eFigure 4 in the Supplement also presents similar RRR adjusted results (at the person level) for other key explanatory factors, including age, sex, health plan benefit type, social deprivation, region of the country, and living in a hot spot state.

## Discussion

Using a very large, well-curated health insurance database, we identified a substantial decrease in the number of in-person ambulatory visits during the first 4 months of the COVID-19 pandemic’s onset, but the use of telehealth services increased dramatically and thus filled some of the gap. Our analyses identified a series of patient, clinical, and geographical factors associated with higher uptake of telehealth, including chronic disease burden, behavioral diagnoses, being an existing patient with a previously diagnosed condition, living in the Northeast, living in urban or affluent neighborhoods, and having health maintenance organization coverage. We also documented the association between COVID-19 prevalence rates in a region and higher telehealth adoption. Among those receiving COVID-19–related care, visits and telehealth use rates were slightly higher, and their medical costs were substantially increased. We also documented that among the study members—both those receiving COVID-19–related services and not—those with 1 or more telehealth contact during the March to June 2020 period had substantially higher per person costs, likely because they represented a more ill group of patients.

### Comparison With Previous Studies

Recently, several other reports and publications have documented telehealth expansion in the COVID-19 era. Some are observational studies^5,23,24,25^ focused on a single health system using electronic health records or survey data. More comprehensive analyses^26^ documented changing national patterns through analyses of electronic health records, or billing data streams collected from cohorts of practitioners and patients in large integrated delivery systems. For instance, using a large health insurance database, FAIR Health^27^ periodically posts a national summary of practitioner billing patterns by month. A recent study^28^ examined telehealth provided by a national panel of several thousand primary care physicians participating in the IQVIA (formerly IMS Health) national disease and therapeutic index audit. A few other recent studies^29,30,31^ also leveraged large claims databases, such as the OptumLabs/United, Castlight Health, and the FAIR Health multipayer database, to study changes in health services use and telehealth adoption. Other recent studies have explored different aspects of COVID-19 era telehealth utilization for selected clinical conditions^32^ and specific patient groups.^33^ The Centers for Disease Control and Prevention^34^ recently reported results relying on data furnished by a sample of telehealth companies. To the best of our knowledge, our study is the first that follows a large, well-defined continuously insured national cohort of patients to offer an assessment of individual, clinical, practitioner, payer, and geographical factors associated with changing patterns of telehealth use at the onset of the COVID-19 pandemic.

### Limitations

Although this study has many strengths, its findings are subject to a series of limitations. This study documented patterns during the initial 4-month phase of the pandemic only. It will be important to continue assessing national telehealth use as virtual care becomes more established, as COVID-19 prevalence increases, and as coverage policies may change. It is likely that the patterns in our study group are not representative of the uninsured or those with Medicare and Medicaid insurance. Although claims data arguably offer a high degree of precision regarding the identification of telehealth services, they may underestimate these services where coding is inadequate. For example, we believe that our 2019 results underestimate the actual number of telehealth services received by this cohort because of more limited coding and coverage at the time. More broadly, any claims-based research is sensitive to missing or inaccurate coding that corresponds to variations in policies or benefit structures across health plans. Our findings related to cost are limited by our focus on allowed insurance charges. We were only able to assess the association of patient factors linked to the data in the health plan’s beneficiary file. For example, we had no racial/ethnic information on the members. Our cohort design included insured persons who were continuously enrolled for 16 months. Persons with uninterrupted coverage may differ from a cross-section of insured US individuals. For example, our inclusion criteria meant that persons dying during this time frame were excluded. Age-specific (pre-COVID-19) national death rates indicate that decedents would likely represent a very small percentage (>.005%) of those in our original database,^35^ but especially when COVID-19–related deaths are considered, our results based only on nondecedents would underestimate total cost and utilization within the health plans, given the high end-of-life expenses.

## Conclusions

The spring of 2020 represented the first time in US history that such a large proportion of Americans had wide access to telehealth services. By undertaking this study, we sought to gain an understanding of the patterns of virtual care during this initial phase of the COVID-19 era. In so doing, it was our intent to identify potential implications for the next phase of the pandemic and thereafter. Our study identified a series of issues worthy of continued research. These include the potential impact of telehealth on protecting patients and practitioners from spread of the virus, an apparent social and geographical digital divide in telehealth access, and the need for a better understanding of cost, utilization, and outcome differences among those receiving and not receiving telehealth. Although some of the associations we uncovered may be unique to the COVID-19 environment, arguably the insights we gained will be relevant to the future trajectory of telehealth no matter what direction it takes. This study can help guide future strategies and actions by policy makers, payers. and professional societies. Specifically, our findings can be used by these parties to assess and modify telehealth barriers and facilitators to maximize value to consumers served by the US health care system during this digital age.

## Notes

Supplement. **eTable 1.** Assessing the Census Region Distribution of the Study Cohort: Estimated Percentage of Each Region’s Under 65 Population Present in This Study’s Research Database **eTable 2. **COVID-19 Cohort Identification Criteria and Study Member Count In the COVID-19 (and Non-COVID) Related Subgroups During the March-June 2020 Study Period **eTable 3.** A Comprehensive List of Telehealth-Eligible Services and Associated Codes **eTable 4.** CMS Designated Codes for Place of Service **eTable 5.** Approach to Designating Telehealth Services (from Among Telehealth Eligible Services) and Type of Modality **eTable 6.** Study Designated Specialty Using CMS Provider Specialty Codes **eTable 7.** Comparison of Age and Chronic Condition Counts for Members with 1+ Telehealth vs In-Person Only Visits in 2020: Broken Down by COVID-19 Related Sub-Group Designation **eFigure 1.** Sample Selection and Subgroup Identification Diagram **eFigure 2.** Ratio of 2020 to 2019 Ambulatory (AMB) Clinical Encounters by Week, March through June **eFigure 3.** Telehealth-Eligible Ambulatory (AMB) Encounters per 1000 Enrollees by Week, March-June 2019 and 2020 **eFigure 4.** Adjusted Relative Risk Ratios (RRRs) Associated With Telehealth vs. In-Person Ambulatory Visits During 2020 COVID-19 Period
