# Supplementary material for: In-Person and Telehealth Ambulatory Contacts and Costs in a Large US Insured Cohort Before and During the COVID-19 Pandemic
Source: JAMA Netw Open. 2021 Mar 23;4(3):e212618. doi: 10.1001/jamanetworkopen.2021.2618 (PMC7988360; doi:10.1001/jamanetworkopen.2021.2618)
Supplement: Supplement. — eTable 1. Assessing the Census Region Distribution of the Study Cohort: Estimated Percentage of Each Region’s Under 65 Population Present in This Study’s Research Database eTable 2. COVID-19 Cohort Identification Criteria and Study Member Count In the COVID-19 (and Non-COVID) Related Subgroups During the March-June 2020 Study Period eTable 3. A Comprehensive List of Telehealth-Eligible Services and Associated Codes eTable 4. CMS Designated Codes for Place of Service eTable 5. Approach to Designating Telehealth Services (from Among Telehealth Eligible Services) and Type of Modality eTable 6. Study Designated Specialty Using CMS Provider Specialty Codes eTable 7. Comparison of Age and Chronic Condition Counts for Members with 1+ Telehealth vs In-Person Only Visits in 2020: Broken Down by COVID-19 Related Sub-Group Designation eFigure 1. Sample Selection and Subgroup Identification Diagram eFigure 2. Ratio of 2020 to 2019 Ambulatory (AMB) Clinical Encounters by Week, March through June eFigure 3. Telehealth-Eligible Ambulatory (AMB) Encounters per 1000 Enrollees by Week, March-June 2019 and 2020 eFigure 4. Adjusted Relative Risk Ratios (RRRs) Associated With Telehealth vs. In-Person Ambulatory Visits During 2020 COVID-19 Period [file jamanetwopen-e212618-s001.pdf]

Supplemental Online Content

Weiner JP, Bandeian S, Hatef E, Lans D, Liu A, Lemke KW. In-person and telehealth ambulatory contacts and costs in a large US insured cohort before and during the COVID-19 pandemic. *JAMA Netw Open*. 2021;4(3):e212618. doi:10.1001/jamanetworkopen.2021.2618

eTable 1. Assessing the Census Region Distribution of the Study Cohort: Estimated Percentage of Each Region's Under 65 Population Present in This Study's Research Database

eTable 2. COVID-19 Cohort Identification Criteria and Study Member Count In the COVID-19 (and Non-COVID) Related Subgroups During the March-June 2020 Study Period

eTable 3. A Comprehensive List of Telehealth-Eligible Services and Associated Codes

eTable 4. CMS Designated Codes for Place of Service

eTable 5. Approach to Designating Telehealth Services (from Among Telehealth Eligible Services) and Type of Modality

eTable 6. Study Designated Specialty Using CMS Provider Specialty Codes

eTable 7. Comparison of Age and Chronic Condition Counts for Members with 1+ Telehealth vs In-Person Only Visits in 2020: Broken Down by COVID-19 Related Sub-Group Designation

eFigure 1. Sample Selection and Subgroup Identification Diagram

eFigure 2. Ratio of 2020 to 2019 Ambulatory (AMB) Clinical Encounters by Week, March through June

eFigure 3. Telehealth-Eligible Ambulatory (AMB) Encounters per 1000 Enrollees by Week, March-June 2019 and 2020

eFigure 4. Adjusted Relative Risk Ratios (RRRs) Associated With Telehealth vs. In-Person Ambulatory Visits During 2020 COVID-19 Period

This supplemental material has been provided by the authors to give readers additional information about their work.

eTable 1. Assessing the Census Region Distribution of the Study Cohort: Estimated Percentage of Each Region's Under 65 Population Present in This Study's Research Database

Region	2018 US Population Under Age 65[1]	Health Plan Members from Which The Study Cohort Was Drawn (Enrolled on March 1, 2020)[a]	Est. % of Region's Under 65 Population in Database[b]
East North Central	38,215,000	7,413,126	19.40%
East South Central	15,480,900	3,735,488	24.13%
Middle Atlantic	33,364,800	6,421,859	19.25%
Mountain	20,216,900	2,766,328	13.68%
New England	11,857,300	2,338,916	19.73%
Pacific	44,496,400	4,009,926	9.01%
South Atlantic	52,376,700	10,366,306	19.79%
West North Central	17,385,600	4,689,088	26.97%
West South Central	33,982,900	7,451,841	21.93%

[a] This column reflects the 50.4 M cohort enrolled on March 1, 2020 from which the study cohort was drawn. See eFigure1. Persons in the cohort with missing state information or residing in non-state US territories not included in this table.

[b] Percentage represents ratio of health plan member count to estimated 2018 US population below age 65.

[1] Kaiser Family Foundation estimates based on the Census Bureau's American Community Survey, 2008-2018. https://www.kff.org/other/state-indicator/distribution-by-age. Accessed October 22,2020.

© 2021 Weiner JP et al. *JAMA Network Open.*

eTable 2. COVID-19 Cohort Identification Criteria and Study Member Count In the COVID-19 (and Non-COVID) Related Subgroups During the March-June 2020 Study Period

A: COVID-19 Cohort Identification Criteria[a]

Code	Description of Code
**(1) COVID-19 diagnosis**	
B9729	Other coronavirus as the cause of diseases classified elsewhere
U071	2019-nCoV Acute Respiratory Disease
**(2) COVID-19 exposure**	
Z03818	Encounter for observation for suspected exposure to other biologic agents ruled out
Z20828	Contact with and exposure to other viral communicable diseases
**(3) COVID-19 screening**	
Z1159	Encounter for screening for other viral diseases
**(4) COVID-19 testing**	
0202U	NFCT DS 22 TRGT SARS-COV-2
0223U	NFCT DS 22 TRGT SARS-COV-2
0224U	ANTIBODY SARS-COV-2 TITER(S)
0225U	NFCT DS DNA&RNA 21 SARSCOV2
0226U	SVNT SARSCOV2 ELISA PLSM SRM
86328	SEVERE ACUTE RESPIRATORY SYNDROME CORONAVIRUS 2 (SARS-COV-2) (CORONAVIRUS DISEASE [COVID-19])
86769	ANTIBODY; SEVERE ACUTE RESPIRATORY SYNDROME CORONAVIRUS 2 (SARS-COV-2) (CORONAVIRUS DISEASE [COVID-19]) (FOR SEVERE ACUTE RESPIRATORY SYNDROME CORONAVIRUS 2 [SARS-COV-2] [CORONAVIRUS DISEASE {COVID-19}] ANTIBODY TESTING USING SINGLE STEP METHOD, USE 86328)
87635	SARS-COV-2 COVID-19 AMP PRB
C9803	HOSPITAL OUTPATIENT CLINIC VISIT SPECIMEN COLLECTION FOR SEVERE ACUTE RESPIRATORY SYNDROME CORONAVIRUS 2 (SARS-COV-2) (CORONAVIRUS DISEASE [COVID-19]), ANY SPECIMEN SOURCE
G2023	SPECIMEN COLLECTION FOR SEVERE ACUTE RESPIRATORY SYNDROME CORONAVIRUS 2 (SARS-COV-2) (CORONAVIRUS DISEASE [COVID-19]), ANY SPECIMEN SOURCE
G2024	SPECIMEN COLLECTION FOR SEVERE ACUTE RESPIRATORY SYNDROME CORONAVIRUS 2 (SARS-COV-2) (CORONAVIRUS DISEASE [COVID-19]), FROM AN INDIVIDUAL IN A SKILLED NURSING FACILITY OR BY A LABORATORY ON BEHALF OF A HOME HEALTH AGENCY, ANY SPECIMEN SOURCE
U0001	CDC 2019 NOVEL CORONAVIRUS (2019-NCOV) REAL-TIME RT-PCR DIAGNOSTIC PANEL
U0002	TO BE USED TO BILL FOR NON-CDC LABORATORY TESTS FOR SARS-COV-2/2019-NCOV (COVID-19)
U0003	INFECTIOUS AGENT DETECTION BY NUCLEIC ACID (DNA OR RNA); SEVERE ACUTE RESPIRATORY SYNDROME CORONAVIRUS 2 (SARS-COV-2) (CORONAVIRUS DISEASE [COVID-19]), AMPLIFIED PROBE TECHNIQUE, MAKING USE OF HIGH THROUGHPUT TECHNOLOGIES AS DESCRIBED BY CMS-2020-01-R.
U0004	2019-NCOV CORONAVIRUS, SARS-COV-2/2019-NCOV (COVID-19), ANY TECHNIQUE, MULTIPLE TYPES OR SUBTYPES (INCLUDES ALL TARGETS), NON-CDC, MAKING USE OF HIGH THROUGHPUT TECHNOLOGIES AS DESCRIBED BY CMS-2020-01-R.

[a] The criteria for designation into the COVID-19 subgroup of the study cohort was having 1+ code within any of the above criteria: (1) diagnosis or (2) exposure or (3) screening or (4) testing. Lists of codes used for each criterium are provided in this table.

COVID-19: Coronavirus Disease 2019, CPT: Current Procedural Terminology, HCPC: Healthcare Common Procedure Coding, ICD-10-CM: International Classification of Diseases, 10th Revision, Clinical Modification.

© 2021 Weiner JP et al. *JAMA Network Open.*

B: Study Member Count In the COVID-19 (and non-COVID) Related Subgroups During the March – June 2020 Study Period (For Cohort Members with 1+ In-Scope Visit)

Subgroup Designation	COVID-19 Category[a]	COVID-19-Related Criteria	Study Members	Study Members Hospitalized	Study Members Not Hospitalized
**Four COVID-19 Related Categories Considered in-Scope[a]**	1	Diagnosis	103,606	7,069	96,537
	2	Exposure	842,042	26,587	815,455
	3	Screening	148,864	9,705	139,159
	4	Testing	376,209	28,409	347,800
**Total COVID-19 Related Subgroup Based on 1+ In-Scope Codes**			1,470,721	71,770	1,398,951
**Not in COVID-19 Related Subgroup**			13,966,497		
[a] See COVID-19 identification criteria listed in 4 separate categories in eTable2-A above					

© 2021 Weiner JP et al. *JAMA Network Open.*

eTable 3: A Comprehensive List of Telehealth-Eligible Services and Associated Codes[a]

Type of Service	CPT and HCPCS Codes associated with Type of Service
**E & M – office**	99201, 99202, 99203, 99204, 99205, 99211, 99212, 99213, 99214, 99215, 99354, 99355, 99421[c], 99422[c], 99423[c], 99441[c], 99442[c], 99443[c], G2012, G2061[c], G2062[c], G2063[c]
**E & M – emergency department [b]**	99281, 99282, 99283, 99284, 99285
**E & M – hospital [b]**	99217, 99218, 99219, 99220, 99221, 99222, 99223, 99224, 99225, 99226, 99231, 99232, 99233, 99234, 99235, 99236, 99238, 99239, 99291, 99292, 99356, 99357, 99468, 99469, 99471, 99472, 99475, 99476, 99477, 99478, 99479, 99480, G0508[c], G0509[c]
**Behavioral health**	0362T, 0373T, 90785, 90791, 90792, 90832, 90833, 90834, 90836, 90837, 90838, 90839, 90840, 90845, 90846, 90847, 90853, 90875, 97151, 97152, 97153, 97154, 97155, 97156, 97157, 97158, G0396, G0397, G0410, G0443, G2086, G2087, G2088
**Rehabilitation**	97110, 97112, 97116, 97150, 97161, 97162, 97163, 97164, 97165, 97166, 97167, 97168, 97530, 97535, 97542, 97750, 97755, 97760, 97761, S9152
**Other**	77427, 90951, 90952, 90953, 90954, 90955 90956, 90957, 90958, 90959, 90960, 90961, 90962, 90963, 90964, 90965, 90966, 90967, 90968, 90969, 90970, 92002, 92004, 92012, 92014, 92507, 92508, 92521, 92522, 92523, 92524, 92601, 92602, 92603, 92604, 94002, 94003, 94004, 94005, 94664, 96110, 96112, 96113, 96116, 96121, 96127, 96130, 96131, 96132, 96133, 96136, 96137, 96138, 96139, 96156, 96158, 96159, 96160, 96161, 96164, 96165, 96167, 96168, 96170, 96171, 97802, 97803, 97804, 98966[c], 98967[c], 98968[c], 98970[c], 98971[c], 98972[c], 99091[c], 99304, 99305, 99306, 99307, 99308, 99309, 99310, 99315, 99316, 99324, 99325, 99326, 99327, 99328, 99334, 99335, 99336, 99337, 99341, 99342, 99343, 99344, 99345, 99347, 99348, 99349, 99350, 99406, 99407, 99446[c], 99447[c], 99448[c], 99449[c], 99451[c], 99452[c], 99453[c], 99454[c], 99457[c], 99458[c], 99473, 99474[c], 99483, 99495, 99496, 99497, 99498, G0108, G0109, G0270, G0296, G0406[c], G0407[c], G0408[c], G0420, G0421, G0425[c], G0426[c], G0427[c],G0436, G0437, G0438, G0439, G0442, G0444, G0445, G0446, G0447, G0459[c], G0506, G0513, G0514, G2010[c], G9685, Q3014[c], T1014[c]

[a] A list of CPT® and HCPCS codes for telehealth-eligible services by type of service [2]

[b] Type of service was excluded from our study.

[c] Code does not require a modifier or place of service code if provided via telehealth.

CPT: Current Procedural Terminology, E & M: means evaluation and management, HCPCS: Healthcare Common Procedure Coding System

[2] CMS List of telehealth services. https://www.cms.gov/Medicare/Medicare-General-Information/Telehealth/Telehealth-Codes. Accessed September 21,2020

© 2021 Weiner JP et al. *JAMA Network Open.*

eTable 4. CMS Designated Codes for Place of Service

Place of Service Category	CMS Place of Service Code	CMS Place of Service Name
**Inpatient**	13	Assisted Living Facility
	14	Group Home
	21	Inpatient Hospital
	31	Skilled Nursing Facility
	32	Nursing Facility
	33	Custodial Care Facility
	51	Inpatient Psychiatric Facility
	54	Intermediate Care Facility
	55	Residential Substance Abuse Treatment Facility
	56	Psychiatric Residential Treatment Center
	61	Comprehensive Inpatient Rehabilitation Facility
**Emergency Department**	23	Emergency Room – Hospital
**Outpatient Facility**	05	Indian Health Service Free-standing Facility
	06	Indian Health Service Provider-based Facility
	07	Tribal 638 Free-standing Facility
	08	Tribal 638 Provider-based Facility
	19	Off Campus-Outpatient Hospital
	22	On Campus-Outpatient Hospital
	24	Ambulatory Surgical Center
	25	Birthing Center
	26	Military Treatment Facility
	52	Psychiatric Facility-Partial Hospitalization
	57	Non-residential Substance Abuse Treatment Facility
	58	Non-residential Opioid Treatment Facility
	62	Comprehensive Outpatient Rehabilitation Facility
	65	End-Stage Renal Disease Treatment Facility
**Office / Clinic**	11	Office
	17	Walk-in Retail Health Clinic
	18	Place of Employment-Worksite
	20	Urgent Care Facility
	49	Independent Clinic
	50	Federally Qualified Health Center
	53	Community Mental Health Center
	60	Mass Immunization Center
	71	Public Health Clinic
	72	Rural Health Clinic
**Telehealth**	02	Telehealth
**Other Ambulatory**	01	Pharmacy
	03	School
	04	Homeless Shelter
	09	Prison/ Correctional Facility
	12	Home
	15	Mobile Unit
	16	Temporary Lodging
	34	Hospice

© 2021 Weiner JP et al. *JAMA Network Open.*

	41	Ambulance - Land
	42	Ambulance – Air or Water
	81	Independent Laboratory
	99	Other Place of Service
	UN	Unknown Place of Service
CMS = Centers for Medicare and Medicaid Services		

© 2021 Weiner JP et al. *JAMA Network Open.*

eTable 5. Approach to Designating Telehealth Services (from Among Telehealth Eligible Services) and Type of Modality

(See text for further explanation of methodology)

A: Telehealth Claims by Modality for March-June 2020 Period

Communication Modality	Claims Count	% of Total
Telehealth (e.g., real-time audio / video)	11,296,298	74.42%
Telephone	1,391,618	9.17%
Telephone or internet	2,898	0.02%
Online digital visit	359,195	2.37%
Asynchronous communication	100,920	0.66%
Remote monitoring	39,824	0.26%
Not specified (Place of Service 02)	1,988,277	13.10%
All modalities	15,179,030	100.00%

B: Assignment of Telehealth Eligible Service Codes to Specific Type of Virtual Visit Modality

**Online digital visit**
98970, 98971, 98972, 99421, 99422, 99423, G2061, G2062, G2063
**Remote monitoring**
99091, 99453, 99454, 99457, 99458, 99473, 99474, G2010
**Video supported telehealth**
G0406, G0407, G0408, G0425, G0426, G0427, G0459, G0508, G0509, Q3014, T1014
**Telephone**
98966, 98967, 98968, 99441, 99442, 99443
**Telephone or internet**
99446, 99447, 99448, 99449, 99451, 99452
**Not specified (in-person or via-telehealth depending on modifiers or place of service codes)**
77427, 90785, 90791, 90792, 90832, 90833, 90834, 90836, 90837, 90838, 90839, 90840, 90845, 90846, 90847, 90853, 90875, 90951, 90952, 90953, 90954, 90955, 90956, 90957, 90958, 90959, 90960, 90961, 90962, 90963, 90964, 90965, 90966, 90967, 90968, 90969, 90970, 92002, 92004, 92012, 92014, 92507, 92508, 92521, 92522, 92523, 92524, 92601, 92602, 92603, 92604, 94002, 94003, 94004, 94005, 94664, 96110, 96112, 96113, 96116, 96121, 96127, 96130, 96131, 96132, 96133, 96136, 96137, 96138, 96139, 96156, 96158, 96159, 96160, 96161, 96164, 96165, 96167, 96168, 96170, 96171, 97110, 97112, 97116, 97150, 97151, 97152, 97153, 97154, 97155, 97156, 97157, 97158, 97161, 97162, 97163, 97164, 97165, 97166, 97167, 97168, 97530, 97535, 97542, 97750, 97755, 97760, 97761, 97802, 97803, 97804, 99201, 99202, 99203, 99204, 99205, 99211, 99212, 99213, 99214, 99215, 99217, 99218, 99219, 99220, 99221, 99222, 99223, 99224, 99225, 99226, 99231, 99232, 99233, 99234, 99235, 99236, 99238, 99239, 99281, 99282, 99283, 99284, 99285, 99291, 99292, 99304, 99305, 99306, 99307, 99308, 99309, 99310, 99315, 99316, 99324, 99325, 99326, 99327, 99328, 99334, 99335, 99336, 99337, 99341, 99342, 99343, 99344, 99345, 99347, 99348, 99349, 99350, 99354, 99355, 99356, 99357, 99406, 99407, 99468, 99469, 99471, 99472, 99475, 99476, 99477, 99478, 99479, 99480, 99483, 99495, 99496, 99497, 99498, 0362T, 0373T, G0108, G0109, G0270, G0296, G0396, G0397, G0410, G0420, G0421, G0436, G0437, G0438, G0439, G0442, G0443, G0444, G0445, G0446, G0447, G0506, G0513, G0514, G2012, G2086, G2087, G2088, G9685, S9152

© 2021 Weiner JP et al. *JAMA Network Open.*

C: Service Modifier and Place of Service Codes Used to Designate Telehealth Service and Modality

Code Type	Code	Definition	Modality Classification
**Modifier**	GT	"Via interactive audio and video telecommunications systems."	Video-Supported telehealth
	GQ	"Via an asynchronous telecommunications system."	Asynchronous Communication
	95	"Synchronous telemedicine service rendered via a real-time interactive audio and video telecommunications system."	Video Supported-Telehealth
**Place of Service**	02	The location where health services and health related services are provided or received, through a telecommunication system.	Telehealth - not specified

© 2021 Weiner JP et al. *JAMA Network Open.*

eTable 6. Study Designated Specialty Using CMS Provider Specialty Codes

Study Designated Specialty	CMS Provider Specialty Code	Specialty Description
**Primary Care**	1	GENERAL PRACTICE
	8	FAMILY PRACTICE
	11	INTERNAL MEDICINE
	16	OBSTETRICS GYNECOLOGY
	37	PEDIATRIC MEDICINE
	38	GERIATRIC MEDICINE
**Medical Specialist**	3	ALLERGY / IMMUNOLOGY
	6	CARDIOLOGY
	7	DERMATOLOGY
	10	GASTROENTEROLOGY
	13	NEUROLOGY
	21	CARDIAC ELECTROPHYSIOLOGY
	29	PULMONARY DISEASE
	39	NEPHROLOGY
	44	INFECTIOUS DISEASES
	46	ENDOCRINOLOGY
	66	RHEUMATOLOGY
	81	CRITICAL CARE (INTENSIVISTS)
	82	HEMATOLOGY
	83	HEMATOLOGY / ONCOLOGY
	84	PREVENTIVE MEDICINE
	90	MEDICAL ONCOLOGY
	C0	SLEEP MEDICINE
**Surgical Specialist**	2	GENERAL SURGERY
	4	OTOLARYNGOLOGY
	14	NEUROSURGERY
	18	OPTHALMOLOGY
	19	ORAL SURGERY (DENTISTS ONLY)
	20	ORTHOPEDIC SURGERY
	23	SPORTS MEDICINE
	24	PLASTIC AND RECONSTRUCTIVE SURGERY
	28	COLORECTAL SURGERY (FORMERLY PROCTOLOGY)
	33	THORACIC SURGERY
	34	UROLOGY
	40	HAND SURGERY
	76	PERIPHERAL VASCULAR DISEASE
	77	VASCULAR SURGERY
	78	CARDIAC SURGERY
	85	MAXILLOFACIAL SURGERY
	91	SURGICAL ONCOLOGY
	98	GYNECOLOGICAL / ONCOLOGY
**Behavioral Health**	26	PSYCHIATRY
	27	GERIATRIC PSYCHIATRY
	62	INDEPENDENT BILLING PSYCHOLOGIST

© 2021 Weiner JP et al. *JAMA Network Open.*

	68	CLINICAL PSYCHOLOGIST
	79	ADDICTION MEDICINE
	86	NEUROPSYCHIATRY
**Rehabilitation**	12	OSTEOPATHIC MANIPULATIVE THERAPY
	15	SPEECH LANGUAGE PATH. IN PRIV. PRACTICE
	25	PHYSICAL MEDICINE AND REHABILITATION
	35	CHIROPRACTIC
	65	INDEPENDENTLY PRACTICING PHYSICAL THERAPIST
	67	INDEPEND. PRACTICING OCCUPATIONAL THERAPIST
**Physician Assistant / Nurse Practitioner**	50	NURSE PRACTITIONER
	89	CERTIFIED CLINICAL NURSE SPECIALIST
	97	PHYSICIAN ASSISTANT
**Other Physician / Practitioner**	5	ANESTHESIOLOGY
	9	INTERVENTIONAL PAIN MANAGEMENT
	22	PATHOLOGY
	30	DIAGNOSTIC RADIOLOGY
	32	ANESTHESIOLOGIST ASSISTANTS
	36	NUCLEAR MEDICINE
	41	OPTOMETRY
	42	CERTIFIED NURSE MIDWIFE
	43	CERTIFIED RN ANESTHETIST, ANESTHESIA ASSISTANT
	48	PODIATRY
	55	INDIVIDUAL CERTIFIED ORTHOTIST
	56	INDIVIDUAL CERTIFIED PROSTHETIST
	57	INDIVIDUAL CERTIFIED PROSTHETIST-ORTHOTIST
	64	INDEPENDENTLY BILLING AUDIOLOGIST
	70	MULTI SPECIALTY CLINIC OR GROUP PRACTICE
	71	REGISTERED DIETICIAN/NUTRITION PROFESSIONAL
	72	PAIN MANAGEMENT
	80	LICENSED CLINICAL SOCIAL WORKER
	92	RADIATION ONCOLOGY
	93	EMERGENCY MEDICINE
	94	INTERVENTIONAL RADIOLOGY
	96	OPTICIAN
	99	UNKNOWN PHYSICIAN SPECIALTY
	B2	PEDORTHIC PERSONNEL
	B5	OCULARIST
**Other**	51	MEDICAL SUPPLY COMP.WITH CERTIFIED ORTHOTIST
	52	MEDICAL SUPPLY COMP. W/CERTIFIED PROSTHETIST
	53	MED SUPPLY CO. W/CERT. PROSTHETIST ORTHOTIST
	54	MEDICAL SUPPLY COMPANY NOT 51, 52, OR 53
	58	MEDICAL SUPPLY COMP. W/REGIST. PHARMACIST
	59	AMBULANCE SERVICE SUPPLIER
	60	PUBLIC HEALTH OR WELFARE AGENCIES
	61	VOLUNTARY HEALTH OR CHARITABLE AGENCIES
	63	PORTABLE X-RAY SUPPLIER
	69	INDEPENDENTLY BILLING CLINICAL LABORATORY
	73	MASS IMMUNIZATION ROSTER BILLER
	75	SLIDE PREPARATION FACILITIES

© 2021 Weiner JP et al. *JAMA Network Open.*

	87	ALL OTHER SUPPLIERS
	88	UNKNOWN SUPPLIER
	A4	HOME HEALTH AGENCY
	A5	PHARMACY
	A6	MEDICAL SUPPLY COMP. W/RESPIRATORY THERAPIST
	A7	DEPARTMENT STORE
	A8	GROCERY STORE
	B3	MEDICAL SUPPLY CO W/ PEDORTHIC PERSONNEL
	B4	REHABILITATION AGENCY
	C1	CENTRALIZED FLU
	IN	INVALID
**Facility**	17	HOSPICE AND PALLIATIVE CARE
	31	INTENSIVE CARDIAC REHABILITATION (ICR)
	45	MAMMOGRAPHY SCREENING CENTER
	47	INDEPENDENT DIAGNOSTIC TESTING FACILITY
	49	AMBULATORY SURGICAL CENTER
	74	RADIATION THERAPY CENTER
	A0	HOSPITAL
	A1	SKILLED NURSING FACILITY
	A2	INTERMEDIATE CARE NURSING FACILITY
	A3	NURSING FACILITY, OTHER

© 2021 Weiner JP et al. *JAMA Network Open.*

eTable 7. Comparison of Age and Chronic Condition Counts for Members with 1+ Telehealth vs In-Person Only Visits in 2020: Broken Down by COVID-19 Related Sub-Group Designation (Referent to Text Table 4[a])

	Not in COVID-19 Related Sub-Group[b]					In COVID-19 Related Sub-Group[b]				
	**Person Count for In-Person Visit Only**	**Person Count for 1+ Tele-health**	**In-person Only, %**	**Tele-health, %**	**Un-adj. RRR[c]**	**Person Count for In-Person Visit Only**	**Person Count for 1+ Telehealth**	**In-person Only, %**	**Tele-health, %**	**Un-adj. RRR[c]**
**Total**	9,591,554	4,374,943	100.0	100.0	1.00	868,249	602,472	100.0	100.0	1.00
**Age**										
0 to 5	584,043	130,774	6.09	2.99	0.49	19,704	10,118	2.27	1.68	0.74
6 to 17	1,226,041	457,350	12.78	10.45	0.82	49,092	26,645	5.65	4.42	0.78
18 to 34	1,958,542	936,927	20.42	21.42	1.05	255,008	146,219	29.37	24.27	0.83
35 to 49	2,266,217	1,173,574	23.63	26.82	1.14	241,772	179,885	27.85	29.86	1.07
50 to 64	3,240,774	1,510,429	33.79	34.52	1.02	278,844	217,414	32.12	36.09	1.12
65+	315,934	165,889	3.29	3.79	1.15	23,829	22,191	2.74	3.68	1.34
**Chronic Condition Count in 2019**										
0	3,779,992	1,038,702	39.41	23.74	0.60	381,361	165,957	43.92	27.55	0.63
1	2,894,288	1,315,993	30.18	30.08	1.00	239,340	161,819	27.57	26.86	0.97
2	1,614,463	948,159	16.83	21.67	1.29	131,186	117,063	15.11	19.43	1.29
3+	1,302,811	1,072,089	13.58	24.51	1.80	116,362	157,633	13.40	26.16	1.95

Abbreviation: RRR, relative risk ratio.

[a] See text Table 4. The above table present information on the case-mix of those persons in the last four columns in that table.

[b] 1+ COVID-19 related diagnosis codes reported for each person from COVID-19 related sub-group. Refer to eTable 2 for more information on logic to identify persons with potential or actual COVID-19 Dx during 2020 study period. These includes people tested and/or treated and/or diagnosed with COVID-19.

[c] These ratios reflect the telehealth users' proportion in each row divided by the non-telehealth users' proportion. For example, the bottom left relative risk ratio of 1.80 indicates that for the non-COVID group, a far higher proportion of telehealth users have 3+ chronic conditions relative to the in-person only members i.e., (24.51 ÷ 13.58). The 1.95 ratio in the same row for the COVID-19 suspect group indicates a very similar trend for this group as well.

© 2021 Weiner JP et al. *JAMA Network Open.*

eFigure1: Sample Selection and Subgroup Identification Diagram

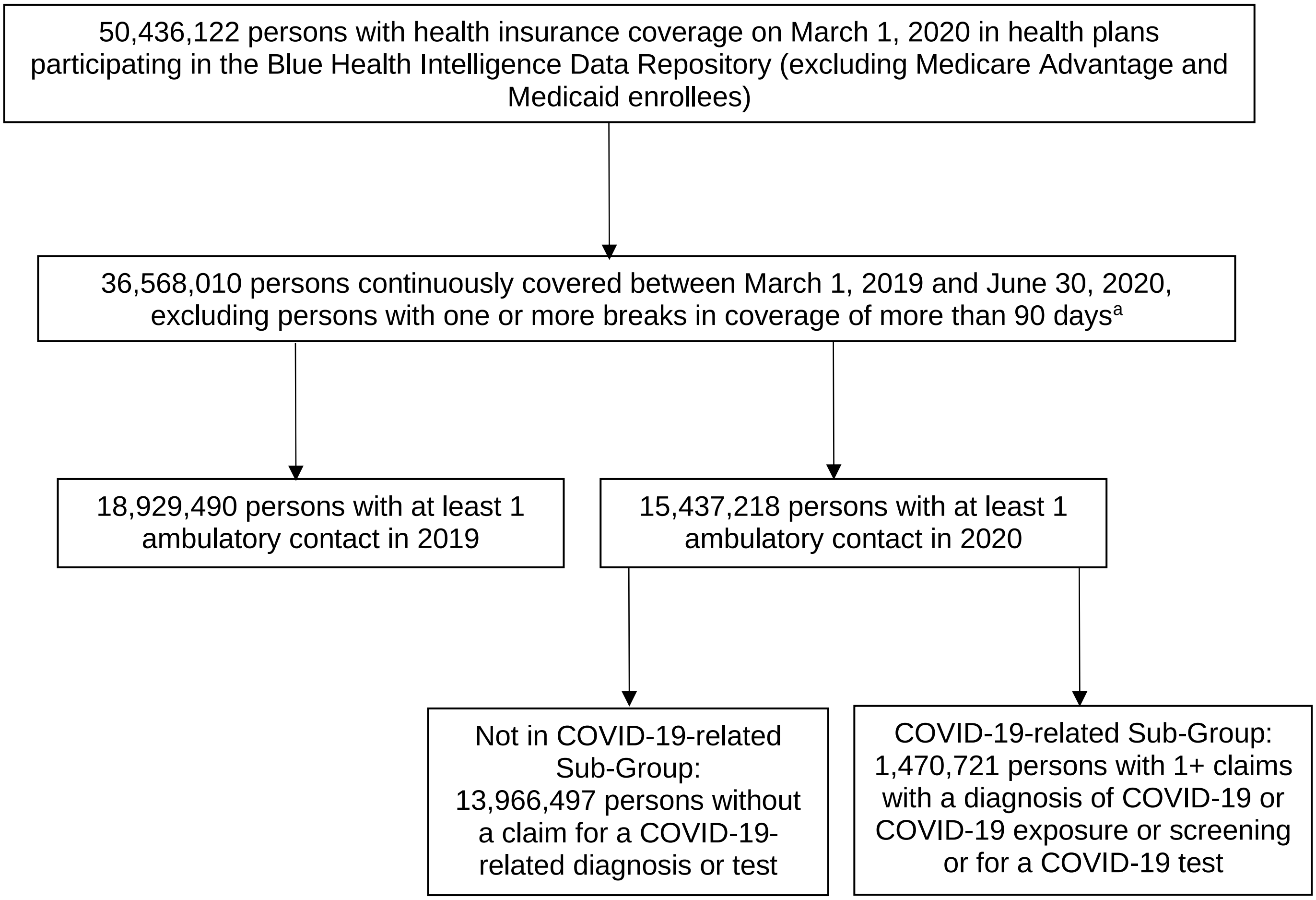

Notes for eFigure 1: Ambulatory contacts are encounters for a set of CPT® and HCPCS codes that in 2020 are eligible for payment on a remote, telehealth basis. See eTable 1 for a listing of codes. Inpatient and hospital emergency department places of service are excluded from the definition, as are service codes that are explicitly defined as inpatient or emergency services. A contact or encounter is defined as a unique combination of member, rendering physician, date of service, and place of service.

Diagnosis codes and service codes for defining the COVID-19-related cohort are in eTable 3. Any claim diagnosis, primary or secondary, on any type of claim, will qualify a person for this cohort, if provided during the 2020 study period; similarly, any claim for a lab test of specimen collection service explicitly defined for COVID-19 will qualify a person for the COVID-19-related cohort.

[a] During this 16-month study period, we allowed up to 90 days for a person to disenroll and re-enroll to account for coverage changes during period. Only 1.2% of cohort had any gap in coverage and of these, the majority of those with a gap (67%) had a gap of one month or less.

© 2021 Weiner JP et al. *JAMA Network Open.*

eFigure 2. Ratio of 2020 to 2019 Ambulatory (AMB) Clinical Encounters by Week, March Through June

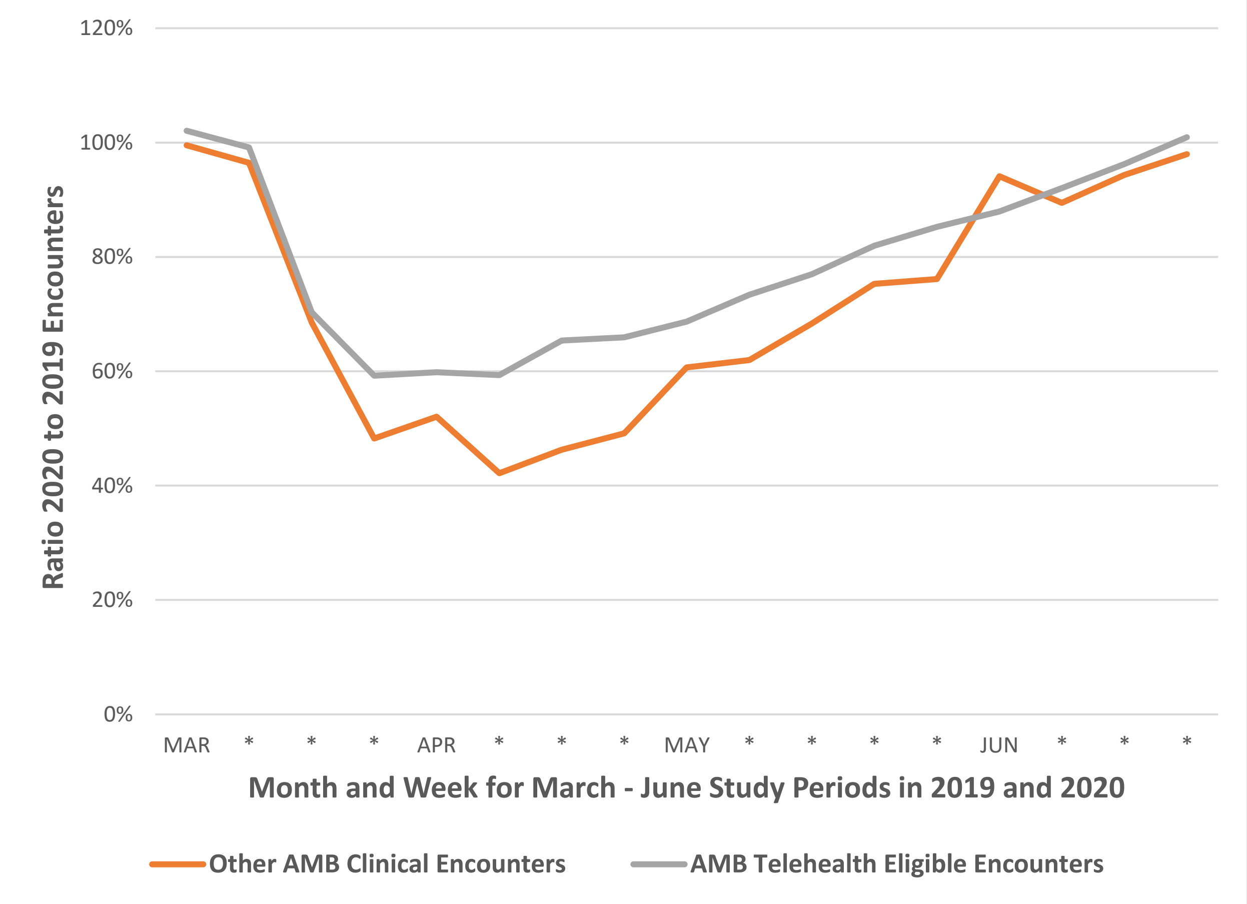

Notes for eFigure 2: The first graph distinguished between "telehealth eligible services" (our list of 264 codes) and all other services provided on an ambulatory basis, except for lab tests and ED visits. Together these two categories correspond to almost all ambulatory care that requires a clinical encounter.
By the second week of April 2020, Other ambulatory (AMB) encounters (orange line) had decreased to only 42% of 2019 levels. In contrast, telehealth eligible services (grey line) were less severely impacted and decreased to 59% of 2019 Levels. By the end of June both categories have steadily climbed back almost to 2019 levels.

The graphs use adjusted encounter counts to account for claim lag using standard actuarial methods.

© 2021 Weiner JP et al. *JAMA Network Open.*

eFigure 3. Telehealth-eligible Ambulatory (AMB) Encounters per 1000 Enrollees by Week, March-June 2019 and 2020

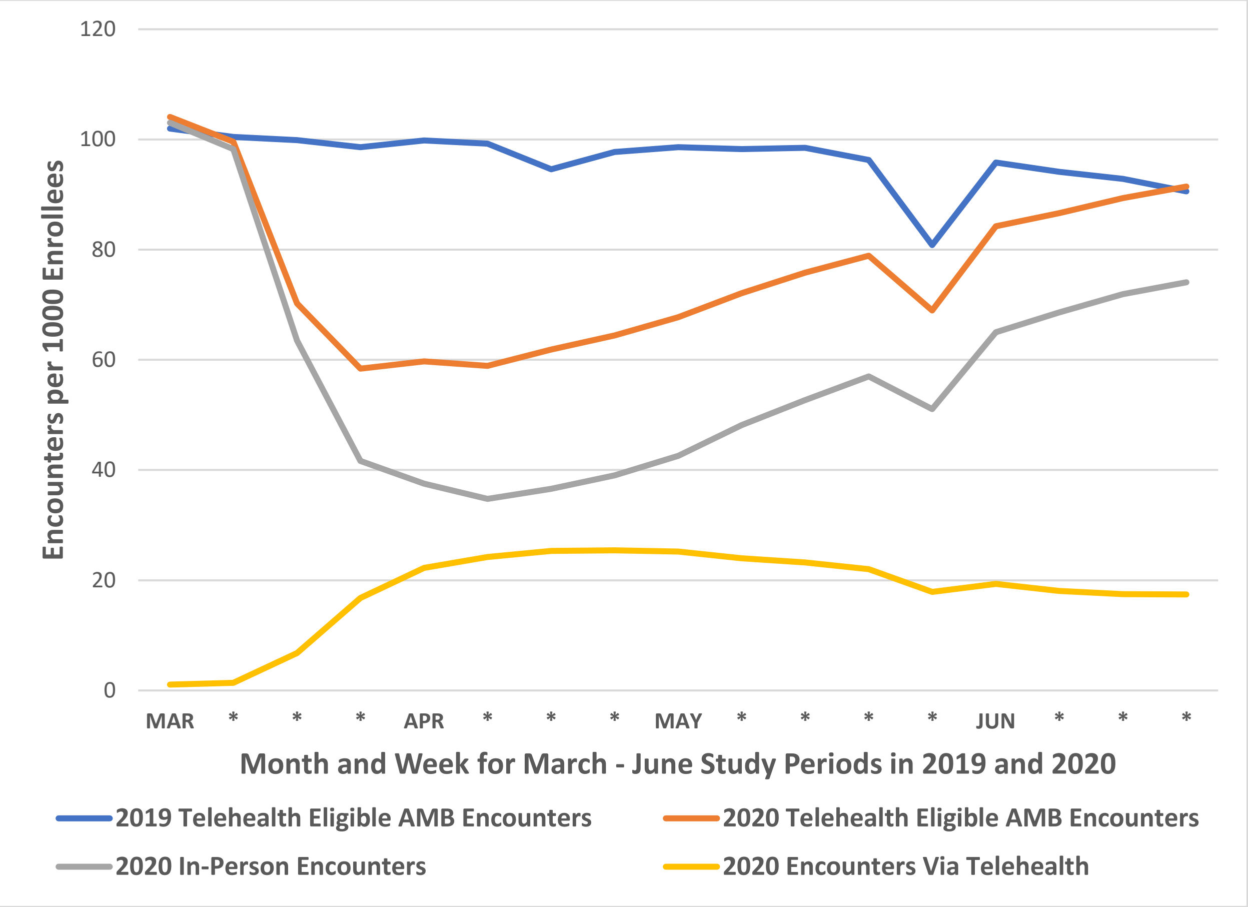

Notes for eFigure 3: The graphs are based on encounters per 1000 enrollees and explain a disparity in impact. This graph focuses exclusively on "telehealth eligible services" and distinguishes between those provided on an "in-person" basis and those provided "via telehealth".

In-person encounters in 2020 were also severely impacted and dropped to a low of 38.2 encounters per 1000, but this drop was partially offset by a sharp increase in telehealth reaching a peak of 25.4 telehealth encounters per 1000 in the 4th week of April before trending down to a rate of 17.4 per 1000 at the end of June. In effect, telehealth played a critical role in helping to ensure continuity and availability of care at the peak of the crisis in the spring of 2020.

The graphs use adjusted encounter counts to account for claim lag using standard actuarial methods.

© 2021 Weiner JP et al. *JAMA Network Open.*

eFigure 4. Adjusted Risk Ratios (RRRs) Associated With Telehealth vs. In-Person Ambulatory Visits During 2020 COVID-19 Period

Note that panels A, B, C, and D below represent expanded versions (including confidence intervals) of the graphs that appear in Figure 1 of the main text. The other RRR charts below do not appear in the text.

RRRs for <u>Encounter-level Model</u> (Based on 46,453,007 Encounters)

A)

Characteristic	RRR (95% CI)
5 (high)	1.335 (1.333,1.337)
4	1.152 (1.150,1.154)
3	1.038 (1.036,1.040)
2 (low)	0.989 (0.988,0.991)

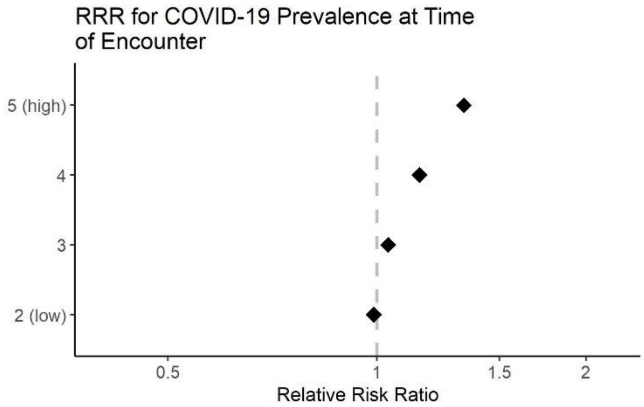

RRR of telehealth contact at encounter level as function of COVID-19 prevalence during week. Area's COVID-19 rates ranked by national quintile. Reference is 1st (lowest) quintile.

B)

Characteristic	RRR (95% CI)
Eye	0.331 (0.329,0.334)
Pregnancy	0.418 (0.413,0.423)
Injury	0.452 (0.449,0.455)
Neoplasms	0.526 (0.523,0.529)
Ear	0.552 (0.549,0.556)
Musculoskeletal	0.665 (0.663,0.667)
Skin	0.676 (0.674,0.679)
Health status	0.831 (0.829,0.834)
Genitourinary	0.833 (0.830,0.836)
Blood and immune	0.861 (0.854,0.868)
Respiratory	0.898 (0.896,0.901)
Infectious	0.949 (0.944,0.954)
COVID-19-related	0.977 (0.972,0.981)
Circulatory	1.020 (1.017,1.023)
Endocrine	1.057 (1.054,1.060)
Congenital	1.122 (1.110,1.134)
Digestive	1.217 (1.213,1.221)
Nervous system	1.281 (1.277,1.285)
Behavioral disorder	1.631 (1.627,1.636)

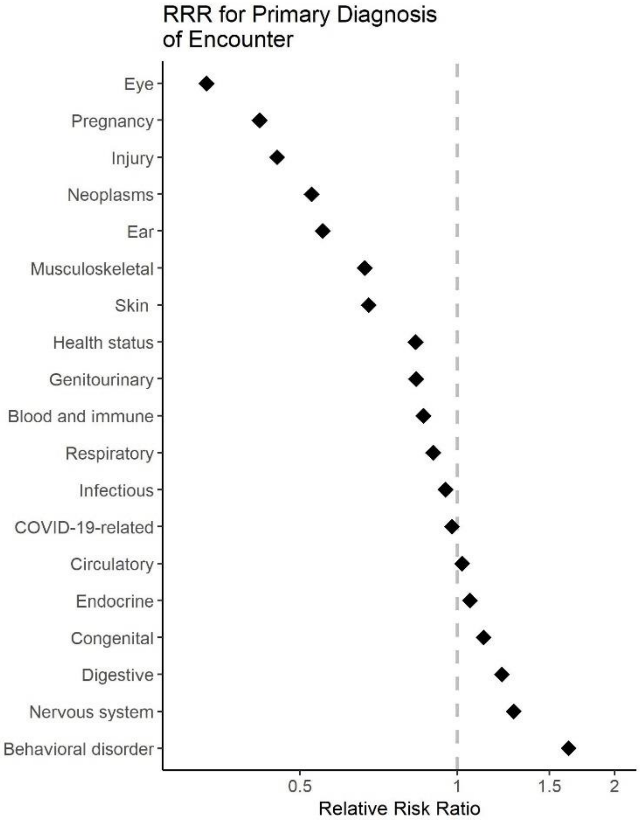

RRR of telehealth contact at encounter level as function of primary problem. Reference is ICD chapter for signs and symptoms.

© 2021 Weiner JP et al. *JAMA Network Open.*

RRRs <u>for Person-level Model</u> (15,437,217 Members with 1+ contacts in this analysis)

C)

Characteristic	RRR (95% CI)
3 or more	2.001 (1.996,2.006)
2	1.664 (1.660,1.667)
1	1.412 (1.409,1.415)

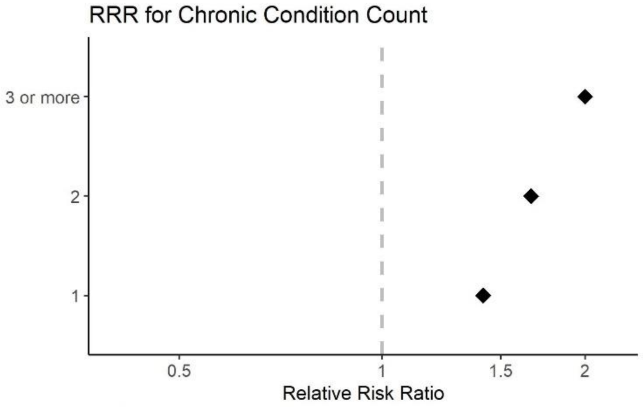

RRR of person with ambulatory services having 1+ telehealth visit as a function of patient's chronic condition count. Reference count is 0.

D)

Characteristic	RRR (95% CI)
Noncore Counties	0.629 (0.627,0.632)
Micropolitan Counties	0.699 (0.697,0.701)
Small Metro	0.743 (0.741,0.745)
Medium Metro	0.826 (0.824,0.828)
Large Fringe Metro	0.952 (0.949,0.954)

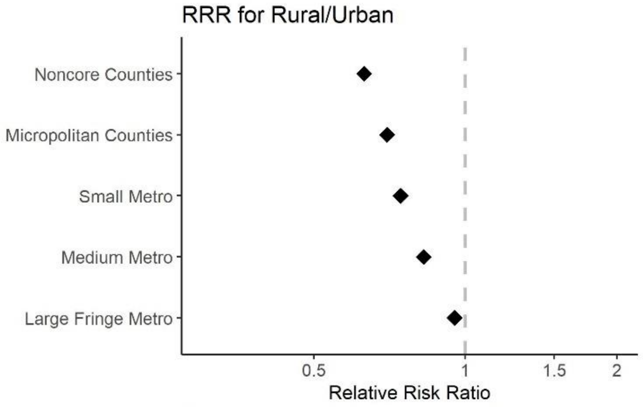

RRR of person with ambulatory services having 1+ telehealth visit as a function of the type of Federal Metropolitan Statistical Area designation of patient's home zip code. Reference is Large Central Metro.

E)

Characteristic	RRR (95% CI)
65+	0.850 (0.847,0.853)
50-64	0.878 (0.877,0.880)
18-34	1.003 (1.001,1.005)
6-17	0.884 (0.882,0.887)
0-5	0.659 (0.656,0.662)

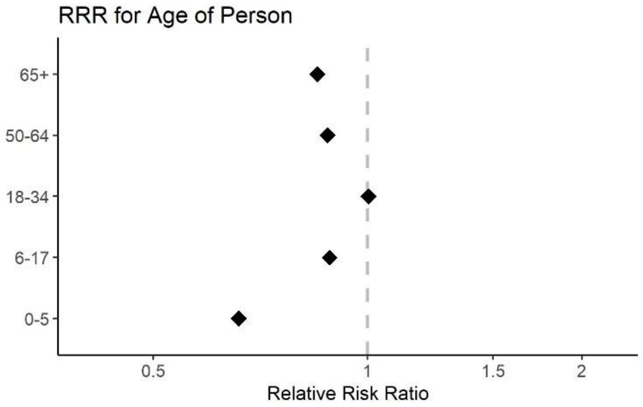

RRR of person with ambulatory services having 1+ telehealth visit as a function of age. Reference is age 35-49.

© 2021 Weiner JP et al. *JAMA Network Open.*

F)

Characteristic	RRR (95% CI)
Female	1.093 (1.091,1.095)

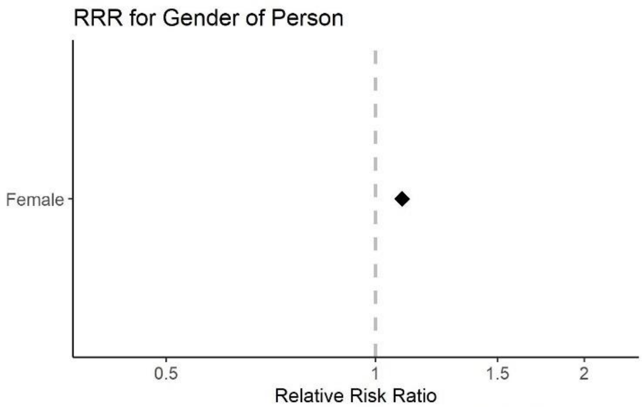

RRR of person with ambulatory services having 1+ telehealth visit as a function of gender. Reference is male.

G)

Characteristic	RRR (95% CI)
HMO	1.181 (1.177,1.184)
High Deductible	0.870 (0.869,0.872)

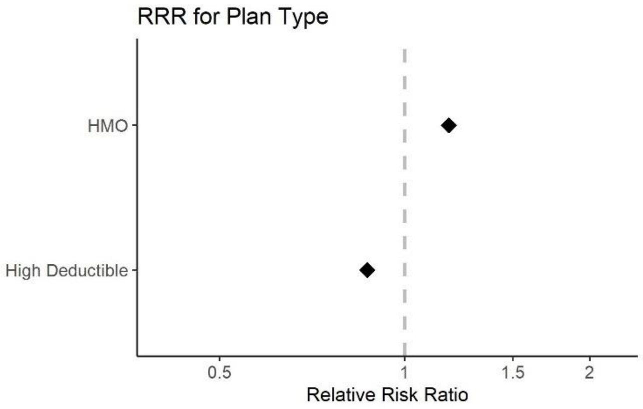

RRR of person with ambulatory services having 1+ telehealth visit as a function of plan type. Reference is standard PPO.

H)

Characteristic	RRR (95% CI)
4	0.940 (0.938,0.942)
3	0.922 (0.920,0.924)
2	0.940 (0.938,0.941)

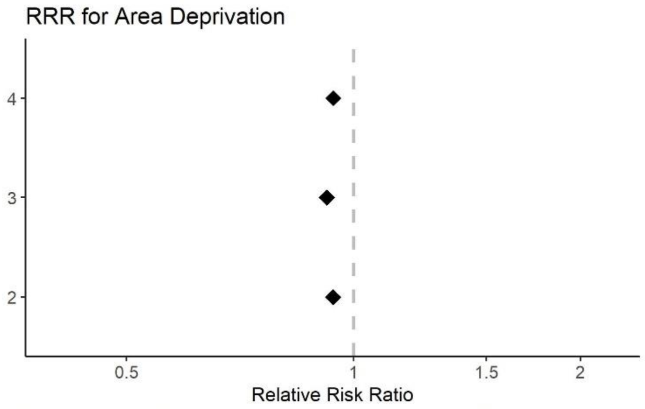

RRR of person with ambulatory services having 1+ telehealth visit as a function of Area Deprivation. Reference is Quartile 1 (least deprived).

© 2021 Weiner JP et al. *JAMA Network Open.*

I)

Characteristic	RRR (95% CI)
West	0.958 (0.955,0.961)
Midwest	0.847 (0.845,0.849)
South	0.840 (0.838,0.842)

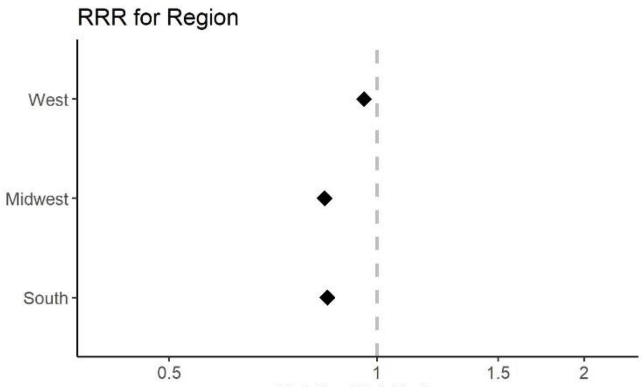

RRR of person with ambulatory services having 1+ telehealth visit as a function of Census Division region. Reference is Northeast.

J)

Characteristic	RRR (95% CI)
Hot Spot	1.225 (1.222,1.228)

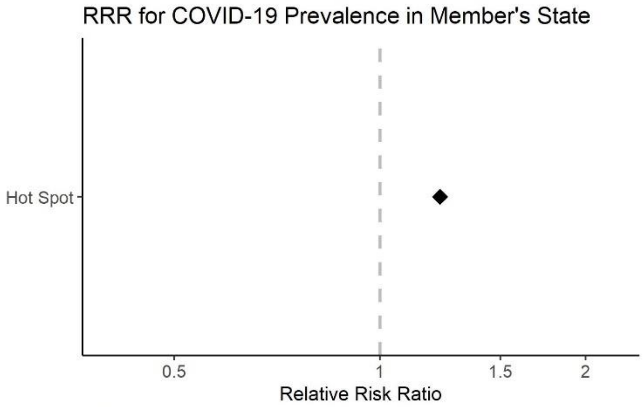

RRR of person with ambulatory services having 1+ telehealth visit as a function of COVID-19 prevalence in State. Reference is not hot spot.

The Relative Risk Ratios (RRR) reflect the odds of a telehealth contact, relative to in-person. The sample included 15,427,217 persons in the continuously insured cohort with 1+ telehealth eligible ambulatory visit during the four-month March-June 2020 study period. These individuals had 46,453,007 in-scope encounters. All risk ratios are adjusted for age, gender, plan type, chronic condition count, morbidity categories, region, urban/ rural, Area Deprivation Index, COVID-19 prevalence during week (for encounter) or “hot spot” (for person level), and type of service. The encounter-level analyses (A and B) are also adjusted for provider specialty and new patient and new condition combinations for that contact. RRR values are plotted on log scales.

© 2021 Weiner JP et al. *JAMA Network Open.*
